# Exploring sensory aspects of cutlery in neurodivergent-informed eating disorder care

**DOI:** 10.3389/fpsyt.2026.1827308

**Published:** 2026-06-11

**Authors:** Dimitri Chubinidze, Adia Meyer, Lauren Makin, Kendal Sterling, Kate Tchanturia

**Affiliations:** 1 Department of Psychological Medicine, Institute of Psychiatry, Psychology and Neuroscience (IoPPN), King’s College London, London, United Kingdom; 2 National Eating Disorders Service, South London and Maudsley National Health Service (NHS) Foundation Trust, London, United Kingdom; 3 Department of Culture and Communication, Aalborg University, Aalborg, Denmark

**Keywords:** ADHD, autism, co-design, eating disorders, mealtime support, neurodiversity-affirming care, PEACE pathway, sensory processing

## Abstract

**Background:**

Sensory sensitivities are common in individuals with eating disorders (ED) particularly among those who are also neurodivergent. However, research has primarily focused on the sensory properties of food and clinical environments, rather than the sensory role of cutlery itself.

**Methods:**

This mixed-methods study examined how adults receiving intensive ED treatment evaluated the sensory properties of cutlery using a structured object-elicitation workshop. The study was conducted within a specialist ED service implementing the autism-friendly PEACE Pathway. Participants evaluated 31 cutlery items varying in material, weight, size, and shape. Quantitative data captured preference rankings and ratings of ease of use, comfort, and sensory pleasantness for preferred items. Qualitative data were generated through written feedback and focus group discussion and were analysed using a framework approach.

**Results:**

Metal cutlery was most frequently preferred, whereas wooden, paper-based, plastic, and hybrid designs (e.g. the spork) were consistently rejected. While participants shared many sensory constraints, there was also marked individual variation. Qualitative findings indicated that cutlery functioned as an active sensory interface, influencing comfort, predictability, and readiness to eat. Judgements were grounded in embodied evaluations of weight, balance, texture, mouth fit, and material neutrality. Preferences remained consistent across treatment contexts, with participants describing coping strategies and sensory compromises rather than changes in sensory preferences.

**Conclusions:**

Cutlery plays an important role in mealtime experience within ED treatment settings. Sensory object elicitation provides a practical method for identifying sensory preferences and translating these into design-relevant insights. These findings support the development of sensory-informed, neurodivergent-affirming adaptations in ED care environments.

## 1 Introduction

Eating is a complex sensory and embodied behaviour. It involves experiencing textures, temperature, sounds, colours and bodily sensations, all of which can contribute to comfort and engagement (1, 2). For individuals with eating disorders (ED), anxiety around mealtimes can heighten sensitivity to these sensory experiences, and aversion can have a significant negative impact on eating. Difficulties with texture, temperature, tactile feedback, and predictability have been reported transdiagnostically across ED presentations and may contribute to distress, food avoidance, and ritualised eating behaviours (1, 3–6). These sensory challenges are particularly salient in individuals with co-occurring autistic or ADHD features, where enduring, multi-domain sensory sensitivities may intensify selective or avoidant eating behaviours, producing ARFID-like presentations, even when ARFID is not the primary diagnosis (7–13). In this paper, we use ‘neurodivergent’ to refer specifically to individuals with autism and/or ADHD, consistent with the scope of the ED care pathway within which the study was conducted (8).

Despite increasing recognition that sensory processing differences in EDs are relevant to treatment engagement and everyday functioning, most research has focused on the food itself or broader eating environments (14). Although cutlery is integral to the act of eating, it is rarely considered as an active contributor to the sensory experience of eating, and therefore its role in comfort and engagement remains relatively unexplored (15). Experimental studies in general populations demonstrate that material properties of cutlery, such as spoon shape or weight, influence perceived quality and enjoyment of food (16–18), suggesting that these seemingly mundane objects can contribute to eating experiences.

In many specialist ED clinics, cutlery is treated as interchangeable, and sensory preferences may be viewed with scepticism by staff or interpreted as ED behaviours rather than accommodated. Patients are typically provided with standardised cutlery, with little attention to individual sensory needs. Research shows that this approach of uniformity and reduced choice can increase distress and reduce feelings of autonomy (14, 19). This standardised approach directly contrasts with sensory-, trauma-, and neurodivergent-informed models of care, which emphasise personalisation and adapting environments to individual needs (14, 20–22). Offering cutlery that aligns with individual sensory preferences, rather than standardised or disposable items, may help patients feel more grounded in everyday routines and reduce feelings of institutional detachment (23). Evidence from occupational therapy and environmental psychology further suggests that even small material or environmental adjustments can meaningfully influence perceived safety, comfort, and engagement in daily activities (20, 22, 24).

The present study was conducted within a specialist ED service implementing the PEACE Pathway (for details www.peacepathway.org), a neurodivergent-informed care framework now adopted across several UK and international ED services. PEACE operationalises neurodivergent-affirming principles through sensory screening at admission, communication adaptions (e.g. communication passports), tailored psychological interventions, and systematic environmental modifications (25). This clinical context provided a suitable setting to examine how sensory needs related to everyday eating tools can be identified and addressed by drawing on patients’ lived sensory experience.

Despite growing evidence highlighting the importance of sensory experiences, empirical research examining how individuals with EDs perceive and interact with the sensory properties of cutlery remains limited. Object-based elicitation methods, which use physical artefacts to prompt reflection and discussion, provide a promising way to capture tacit, embodied knowledge that is difficult to verbalise (26, 27). Applying such methods to cutlery could generate insights to support sensory-informed interventions and design adaptations that improve comfort, predictability, and engagement during mealtimes.

The present study examines how adults in ED treatment programmes evaluate the sensory properties of cutlery through structured object elicitation. By combining handling tasks, preference capture, and qualitative feedback, it aims to:

1. Identify patterned sensory constraints and priorities in cutlery evaluation;
2. Distinguish shared areas of aversion from individual variation; and
3. Generate design insights to inform future prototyping of sensory-friendly cutlery for ED treatment.

## 2 Material and methods

### 2.1 Study design

We used a sensory object-elicitation workshop to examine cutlery preferences among adults undergoing ED treatment. The study design combined structured handling tasks with group discussion and quantitative components. The approach was qualitatively driven, using objects as prompts to elicit embodied sensory judgements, while descriptive quantitative data were used to summarise preferred patterns across the stimulus set.

The workshop was delivered four times with separate groups of 5–7 participants. Each session followed the same 60-minute structured format and was conducted in the clinic.

### 2.2 Participants and setting

Twenty-four participants took part across four workshops. All were patients receiving intensive ED treatment (inpatient or day programme) within a specialist service implementing the PEACE Pathway. Of the 24 participants, six (25%) had a formal neurodevelopmental diagnosis (autism *n* = 3; ADHD *n* = 3). The study group was predominantly White and female. Full demographic and clinical characteristics, including age, gender, ED diagnosis, BMI, illness duration, and comorbid conditions, are reported in **Table 1**. Eligibility criteria included capacity to provide informed consent and willingness to participate in a group workshop. Recruitment was conducted through posters displayed on the wards and announcements at community meetings.

**Table 1:** Summary of participants’ health and demographic characteristics.

Variable	n (%) or M (SD)	Median (IQR)	Min - max
Age	30.71 (15.00)	26.00 (21.00 – 33.75)	18 – 69
Gender, n (%)			
Female	22 (91.6%)		
Male	1 (4.2%)		
Non-binary	1 (4.2%)		
Ethnicity, n (%)			
White British	22 (91.6%)		
White Irish	1 (4.2%)		
White Other	1 (4.2%)		
Diagnosis, n (%)			
AN restrictive subtype	20 (79.1%)		
AN binge-purge subtype	3 (16.7%)		
Bulimia nervosa	1 (4.2%)		
Duration of ED in years		9.50 (4.25 – 12.00)	2 – 20
Missing, n (%)	4 (16.67%)		
BMI on admission	16.02 (2.13)	15.70 (15.13 – 17.60)	12.8 – 21.2
Comorbidity, n (%)			
Anxiety and mood-related comorbidity	13 (54.2%)		
OCD	1 (4.2%)		
OCPD	1 (4.2%)		
EUPD	2 (8.3%)		
Autism	3 (12.5%)		
ADHD	3 (12.5%)		

N, number of participants; SD, standard deviation; ED, eating disorder; BMI, body mass index; AN, anorexia nervosa; OCD, obsessive–compulsive disorder; OCPD, obsessive–compulsive personality disorder; EUPD, emotionally unstable personality disorder; ADHD, attention-deficit/hyperactivity disorder.

The workshops took place after participants had attended the Sensory Wellbeing Group, a clinician-led intervention routinely delivered within the day service and inpatient programme (21, 28). Most participants had attended the Sensory Wellbeing Group at least once while in treatment, although attendance was not an eligibility requirement. Prior attendance meant that participants shared a basic vocabulary for discussing sensory experience (e.g. touch, weight, texture, and temperature), which supported engagement with the elicitation tasks. Sessions were held in a room within the ED service with adjustable lighting and seating arranged in a U-shape or around a single table to maximise visibility and choice.

### 2.3 Stimulus set

**Figure 1:**
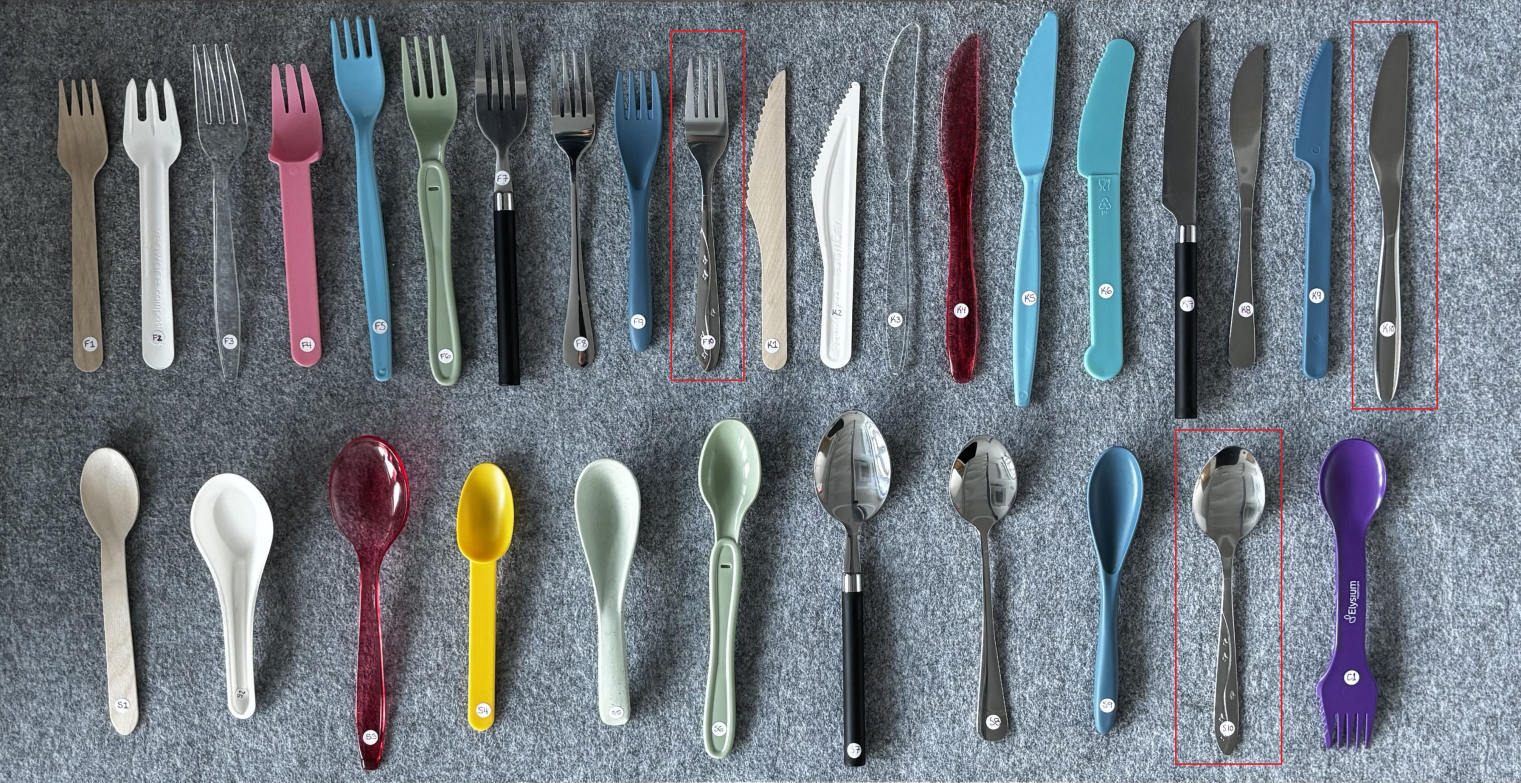
Cutlery stimulus set used in the workshops. The set comprised 31 items (10 forks, 10 spoons, 10 knives, and one spork) varying in material, weight, handle shape, finish, and size. Items outlined in red indicate cutleries most frequently selected among participants’ Top-3 preferences in descriptive quantitative analysis.

The stimulus set comprised 31 cutlery items: 10 forks, 10 spoons, 10 knives, and one spork (**Figure 1**). Items were purposively selected to reflect cutlery commonly available in everyday contexts, including retail, hospitality, hospital canteens, and clinical settings. The set varied across material, weight, handle shape, finish, size, and visual appearance. The number of items was selected to provide sufficient variation across material and shape while remaining manageable within a 60-minute group session. Each item was labelled with a simple alphanumeric code indicating cutlery type and index number. Labels were positioned to avoid interfering with grip. The stimulus set was treated as a prompt for reflection rather than an exhaustive catalogue of possible designs.

### 2.4 Workshop procedure

The workshop followed a structured sequence. First, participants were welcomed, the study was explained, and consent was obtained.

Participants then received a sheet listing all cutlery items and were free to handle the items in any order. They recorded an initial evaluation for each item using simple *Like*, *Unsure*, or *Dislike* categories, with the option to add a brief written reason (1–2 words).

After this, participants selected their Top-3 preferred items and Least-1 preferred item. For each selected item, participants listed three characteristics influencing their choice, such as weight, texture, grip, or shape. Participants also identified a single favourite item from within their Top-3 selections. For the Top-3 items only, participants provided numerical ratings (0-10) for ease of use, comfort, and sensory pleasantness.

Finally, following individual tasks, a whole-group discussion was facilitated. Prompts focused on what made favourite items preferred, what created discomfort in least-preferred items, whether preferences changed across contexts, language used to describe good and poor items, material preferences and avoidances, and desired design changes. Discussions were audio-recorded and supplemented by facilitator field notes and photographs of annotated boards.

### 2.5 Data sources

Data sources included completed activity sheets (initial evaluations, Top-3 and Least-1 selections, ratings, and written rationales), audio recordings of group discussions, facilitator field notes, and photographs of workshop artefacts.

### 2.6 Quantitative analysis

Initial *Like*, *Unsure*, and *Dislike* responses were used to contextualise participants’ written rationales and were not treated as comparative ranking measures. Preference rankings were derived from Top-3 and Least-1 selections.

**Figure 2:**
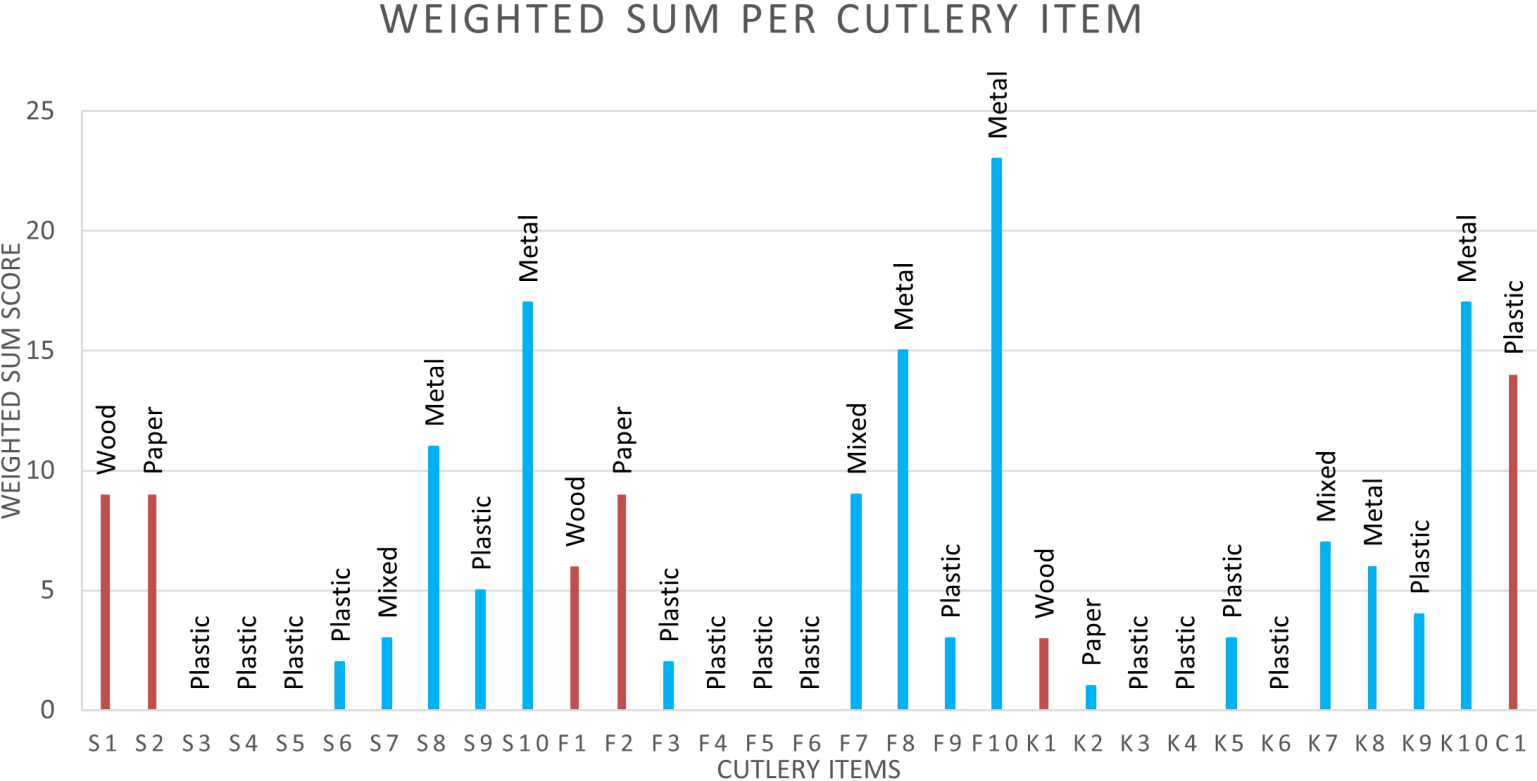
Visual representation of preference rankings for each cutlery item demonstrated by weighted sum. Red bars indicate least favoured items, while blue bars indicate most favoured items.

Counts and ordinal summaries were used to describe preference patterns across items, materials, and cutlery types. For visual comparison (**Figure 2**), a weighted score was also calculated for each item, with Top-3 selections weighted +1 and Least-1 selections weighted −1. For ease of use, comfort, and pleasantness ratings, medians and interquartile ranges were calculated for the Top-3 items only. Given the exploratory and formative nature of the study, no inferential statistical analyses were conducted, and findings are presented descriptively.

### 2.7 Qualitative analysis

Qualitative data were analysed using a framework approach (29, 30). This method was selected because it supported systematic analysis of data generated through structured elicitation, while remaining closely tied to participants’ descriptions of specific objects and features. Data drawn into the analysis comprised both group discussion transcripts and written feedback recorded on the activity sheets, including the brief attribute descriptions participants used to justify their Top-3 and single favourite selections.

Following Gale et al. (29), analysis proceeded through five stages: (1) familiarisation with transcripts, written rationales, and photographs of workshop artefacts; (2) development of an initial analytic framework informed by the workshop prompts (sensory fit, discomfort, context-dependent preferences, language use, material preferences, and desired design changes); (3) indexing of all data to this framework; (4) charting indexed data into matrices organised by participant and theme; and (5) interpretation focused on identifying patterned sensory evaluations while preserving individual variation. The reporting of findings (Section 3.2) follows the structure of this initial framework, with one subsection dedicated to each of the six elicitation domains.

Coding was conducted by DC. Emerging interpretations were discussed with AM to support reflexive examination of assumptions and refinement of analytic categories. Analysis prioritised comparison across participants while retaining attention to individual variation and concrete sensory descriptions, supporting integration with descriptive quantitative findings and design translation.

### 2.8 Ethical considerations

Participation was voluntary, with opt-out options at each stage. The study received South London and Maudsley NHS clinical governance approval (ID 788/17.6.25). All cutlery items were cleaned prior to handling. No sharp blades were included. Data were anonymised using study codes, with linkage files stored separately on secure systems.

## 3 Results

### 3.1 Quantitative results: cutlery preference patterns

Quantitative data were used to describe overall patterns of cutlery preference across participants. Rankings and item-level ratings were examined descriptively to identify distributional trends rather than to test statistical differences.

Across the sample, metal cutlery items accounted for 46 of the 69 Top-3 rankings (66.7%) and only 1 of the 9 Least-1 rankings. In contrast, wooden and paper-based items received no Top-3 rankings and accounted for 12 of the 18 Least-1 rankings for non-metal materials (**Table 2**). Plastic items showed mixed evaluations, with some ranked among preferred items and others appearing among least preferred.

**Table 2:** Descriptive summary of cutlery preference rankings and ratings by material and cutlery type.

Cutlery type	Material	Cutlery		Top 3 ranking (n)	Least-1 rankings (n)	Total rankings (n)	Median comfort score	Median ease score	Median pleasantness score
Spoon	Wood		S1	0	3	3			
	Paper		S2	0	3	3			
	Plastic		S3	0	0	0			
	Plastic		S4	0	0	0			
	Plastic		S5	0	0	0			
	Plastic		S6	1	0	1	8	8	7
	Mixed		S7	3	1	4	8	6	7
	Metal		S8	6	0	6	7.5	8	7.5
	Plastic		S9	3	0	3	9	9	8
	Metal		S10	9	1	10	8	8	8
	*Total*			*22*	*8*	*30*	*8*	*8*	*8*
Fork	Wood		F1	0	2	2			
	Paper		F2	0	3	3			
	Plastic		F3	1	0	1	7	10	8
	Plastic		F4	0	0	0			
	Plastic		F5	0	0	0			
	Plastic		F6	0	0	0			
	Mixed		F7	5	0	5	8	8	8
	Metal		F8	6	0	6	7	7	7
	Plastic		F9	1	0	1	10	10	9
	Metal		F10	12	0	12	7.5	8	8
	*Total*			*25*	*5*	*30*	*7.5*	*8*	*8*
Knife	Wood		K1	0	1	1			
	Paper		K2	0	0	0			
	Plastic		K3	1	0	1	7	10	8
	Plastic		K4	0	0	0			
	Plastic		K5	1	0	1	9	9	7
	Plastic		K6	0	0	0			
	Mixed		K7	5	0	5	8	8	8
	Metal		K8	4	0	4	7	8	7.5
	Plastic		K9	2	0	2	4	4	4.5
	Metal		K10	9	0	9	8	8	8
	*Total*			*22*	*1*	*23*	*7*	*8*	*8*
Spork	Plastic		C1	2	7	9	8	8.5	8.5
	*Total*			*2*	*7*	*9*	*8*	*8.5*	*8.5*
Material Type				Top 3 Rankings (n)	Least -1 Rankings (n)	Total Rankings (n)			
Wood				0	6	6			
Paper				0	6	6			
Plastic				12	7	19			
Mixed				13	1	14			
Metal				46	1	47			

Preference rankings varied across participants, with no single item emerging as universally preferred. This variability was most evident for spoons, which showed a wider distribution of rankings than forks or knives. Forks tended to cluster toward either preferred or non-preferred categories (**Table 2**). The spork consistently appeared toward the lower end of the rankings.

Item-level ratings of ease of use, comfort, and pleasantness were collected for participants’ Top-3 items only.

Overall, descriptive patterns indicated a strong preference for metal cutlery and consistent rejection of wooden or paper-based items, and the spork, alongside substantial individual variation in ranking profiles.

### 3.2 Qualitative results: sensory evaluation of cutlery

The qualitative findings are presented across six subsections, organised around the elicitation prompts used during the workshops. Quotations are identified by focus group: D1 and D2 refer to day programme groups, and I1 and I2 refer to inpatient groups.

#### 3.2.1 Sensory fit and functional adequacy of preferred items

Participants described preferred cutlery in terms of immediate bodily fit, often focusing on how an item felt when held rather than listing individual features. This orientation was also reflected in their behaviour during the workshops, where most participants handled the cutlery in a use-oriented way (i.e. by the handle), indicating that evaluation was grounded in embodied interaction. When explaining Top-3 or favourite selections, participants often began with this immediate sense of fit. One participant explained that preference is instantly recognisable, noting “you will feel it when it’s wrong or right” (D1). This sense of fit was grounded in physical interaction with the object.

Weight and texture were repeatedly identified as key features. Participants described preferred items as feeling balanced and manageable in the hand. As one participant explained, “when it feels right in your hand, it just works” (D2). Items perceived as “too light” or “flimsy” (I1) were generally rejected.

Functional details were also important, particularly for forks. Participants paid attention to prong length and sharpness. One participant described a preferred fork as “light, with a good prong length, and sharp” (I2). Metal cutlery was often associated with reliability and functionality. As one participant commented, “the metal feels like it’s doing what it’s supposed to do, but the plastic … it feels like it’s not going to do what it needs to do because it’s just weak” (D1). Another noted, “I feel like if I was to use one of those plastic forks, I’d end up snapping it” (D1). Alongside the features that defined preferred items, participants spoke with equal clarity about the items they actively rejected.

#### 3.2.2 Sources of discomfort

Discomfort was described in strong and often categorical terms. Wooden cutlery was universally disliked. One participant stated, “Wood is always a no” (I2). This dislike was not limited to use but extended to anticipation and imagined interaction.

Participants described visceral reactions to disliked items, including sensations of itchiness, dryness, and lingering discomfort. One participant said, “It makes my skin crawl” (I2). Another described an aversion that persisted beyond physical contact: “I can still feel it on my fingers even though I’m not touching it” (I2).

Some items were perceived not only as uncomfortable but also as fundamentally inappropriate. Describing wooden cutlery, one participant said: “It just feels like an accident can happen with it. It looks like it could injure me … It’s also dry in your hand. It’s not just bad, it’s wrong” (D1). Oversized spoons were another frequent source of discomfort. One participant captured this directly: “That spoon is massive. It doesn’t even fit in my mouth” (D2). These reactions were not confined to the workshop setting; participants described the same preferences and aversions as persisting across the different contexts in which they ate.

#### 3.2.3 Stability of preferences and context

Participants consistently described their preferences as stable across eating contexts (e.g. inpatient and day programme settings, home, restaurants, cafes, and travel). Rather than changing what they liked, they described changing how they coped when preferred options were unavailable. As one participant mentioned, “context doesn’t change what I prefer, it just changes how I cope” (D1). Another participant described carrying their own cutlery when eating away from home as a preferred strategy for avoiding non-preferred items: “I’d rather bring it myself” (I2).

Using unfamiliar cutlery was described as altering the eating experience itself. One participant explained that eating with a different spoon “changes the whole trajectory of my experience” (I2). For this reason, many participants expressed a strong preference for bringing their own cutlery when possible.

This desire to retain preferred cutlery reflected both the positive sensory fit of familiar items and the active avoidance of negative sensory experiences associated with non-preferred items. Participants described wooden and plastic cutlery not merely as unfamiliar but as “bad” or “wrong”. One participant captured the functional consequence directly: “For a proper meal, if the cutlery feels wrong, that’s it, I can’t eat” (I1). Although their preferences were stable, the language participants used to express them varied, drawing on two distinct registers described next.

#### 3.2.4 Language used to express sensory judgement

Two distinct registers of language were identified through analysis of how participants justified their Top-3 and favourite selections. The first was evaluative shorthand: participants rarely articulated preferences in detailed sensory terms, instead relying on brief, immediate judgements. Phrases such as “it just feels right” or “you just kind of know” were common (I2), reflecting certainty without explanation. As one participant put it: “It’s just goes right or it goes wrong” (D1). This was especially noticeable when participants were asked to justify favourite or Top-3 selections, where brief phrases often replaced detailed description.

The second register drew on more specific sensory descriptors. Terms such as “flimsy” (D1), “uneven” and “weird” (D2), and “bendy” and “scratchy” (I2) referred to identifiable physical properties, including flexibility, surface irregularity, texture, and size. These features directly affected handling and mouth fit. Some descriptions combined shape and judgements, as when one participant described an oversized spoon: “This one looks like a shovel. You can’t scrape yoghurt out with that” (D2). Together, these registers allowed participants to communicate embodied judgements while pointing to concrete material features.

#### 3.2.5 Material hierarchies and sensory consequences

Participants articulated a clear hierarchy of materials. Metal was consistently preferred and was associated with cleanliness and reliability. One participant stated simply, “Metal is the cleanest” (I2).

In contrast, plastic and wood were associated with contamination and sensory disruption. One participant described plastic as absorbent: “Plastic absorbs” (I2). Wood was linked to taste transfer and dryness: “I don’t want to eat off wood. I don’t want to taste it. If you make a cup of tea with a wooden stirrer, it tastes like wood” (D2) and another participant said: “I hate that, because the wood comes through into the water” (I1).

Participants also contrasted material properties in terms of weight, noting that “you can’t really get the same weight with another material” (I2), framing non-metal materials as lacking the solidity needed for confident use.

Across accounts, metal was described as offering consistency, appropriate weight, and ease of cleaning. As one participant summarised: “Metal is the priority: cleanliness, consistency, and not being too big. It still has to have a purpose though. It needs some weight in your hand—not too light, not flimsy” (I1). These qualities were prioritised over appearance, positioning materiality as central to sensory confidence during eating.

#### 3.2.6 Desired feature-level modifications

When discussing improvements, participants focused on specific features rather than cutlery items as a whole, emphasising refinement over wholesale redesign. As one participant put it: “It’s not even the whole utensil, it’s specific features. The size of the prongs, the depth of the spoon. That’s what needs changing” (D2). Suggested changes included removing ridges, reducing seams, and making handle thickness more consistent. These requests were often framed in relation to favourite items, with participants describing what would make a preferred option fully usable.

Spoons were particularly important. One participant stated, “I care mostly about the spoon” (I2), linking this to the greater degree of mouth contact involved: “more of it goes in your mouth, it’s more intimate” (I2). Another participant echoed this: “I can deal with a bad fork or knife, but a bad spoon really bothers me … The deep ones are uncomfortable and kind of intimidating—you have to put the whole thing in” (I1). Participants repeatedly referred to teaspoons as an ideal size. One described it simply: “My preference is always a teaspoon” (I2). Smaller, shallower spoons were perceived as less intrusive and easier to tolerate. Taken together, these accounts indicate that cutlery functioned as more than a passive utensil during eating in ED treatment, with participants’ evaluations grounded in stable, feature-level sensory judgements. The implications of these findings for sensory-informed ED care and for future design work are considered in the Discussion that follows.

## 4 Discussion

This study examined how adults receiving ED treatment evaluated cutlery through direct sensory engagement. The findings indicate that cutlery was not experienced as a neutral or interchangeable tool, but as an active mediator of eating experience. Participants’ accounts showed that cutlery could both support or disrupt eating, independent of food properties. This was evident in the strength of negative reactions to certain items and in the care and specificity with which preferred items were described.

These findings extend existing work on sensory sensitivity and eating-related distress (1, 2, 12, 14) by highlighting the role of non-food objects in shaping eating experiences. Prior research has focused primarily on food texture, taste, and olfactory cues (9, 13), as well as environmental factors such as noise and visual stimulation (12, 14, 20). The present study suggests that eating-related tools themselves form part of the sensory landscape of eating, influencing how meals are anticipated, enacted, and tolerated.

### 4.1 Shared sensory constraints alongside individual variation

Quantitative findings showed substantial individual variation in preference rankings, particularly for spoons. At the same time, clear avoidance patterns emerged for specific materials and designs, most notably wooden cutlery and hybrid items such as sporks. Qualitative analysis helps to explain this tension between variation and consistency.

While there were no universally preferred items, there were clearer areas of shared dislike. Certain materials and designs were consistently rejected, indicating common sensory limits across participants. Among preferred items, variation was observed in features such as balance, weight, and shape. Quantitative clustering of least-preferred items reflects these shared constraints, whereas variation among preferred items reflects individual differences within an acceptable sensory range.

This distinction is important for both interpretation and design translation. Rather than aiming to optimise for an average preference, design efforts may be better directed toward respecting shared sensory boundaries while allowing flexibility within them.

### 4.2 Feature-level evaluation with a focus on spoons

Participants evaluated cutlery primarily at the level of specific features rather than whole objects. Weight distribution, handle thickness, surface continuity, prong shape, and spoon depth were discussed more frequently than overall appearance. Among cutlery types, spoons emerged as particularly important.

Quantitatively, spoons showed greater variability in preference rankings than forks or knives. Qualitatively, spoons attracted the strongest affective language and the most detailed critique. Participants repeatedly described spoon size, depth, and fit as critical, often contrasting oversized or deep spoons with the familiarity and tolerability of teaspoons.

This emphasis appears linked to the degree of bodily intimacy involved. Participants described spoons as entering the mouth more fully than other forms of cutlery, increasing sensitivity to shape, depth, and surface features. As a result, spoon design appeared less forgiving, with narrower margins for acceptable variation. However, preference for smaller spoons may also reflect ED-related concerns, such as perceptions of portion size, the amount of food carried per bite, or magical thinking that links smaller utensils to reduced caloric intake or symbolic restraint (31, 32), although these interpretations were not explicitly articulated by participants during the study.

These findings suggest that spoons may warrant particular attention in future design work. Feature-level refinement, rather than wholesale redesign, may be especially important for this cutlery type.

### 4.3 Material hierarchies and sensory predictability

Participants articulated a clear hierarchy of materials, with metal consistently preferred and wood and paper most strongly rejected. Plastic occupied an intermediate position, tolerated in constrained contexts but rarely preferred. These hierarchies were grounded in concerns about predictability, hygiene, and sensory carryover rather than aesthetics.

Wooden cutlery was associated with dryness, texture interference, taste transfer, and lingering sensory memory. For wood, participants described aversion that extended beyond direct contact to anticipation of the sensations associated with use. Plastic was associated with lightness and flexibility, sometimes perceived as unreliable or insufficient for what one participant described as a “proper meal” (I1). Conversely, metal was valued for its weight, durability, ease of cleaning, and perceived neutrality.

These findings align with work on anticipatory disgust (33–35), sensory memory, and material perception (36). Although participants in this study varied in neurodivergent profiles, the findings also resonate with autism-informed design principles emphasising predictability and consistency (8). In the present sample, 12.5% of participants had a recorded autism diagnosis, and 12.5% had ADHD, although sensory preferences were not confined to these groups.

Importantly, context appeared to influence coping strategies rather than changing core preferences. Participants described tolerating less-preferred materials in settings such as flights or clinical environments, but this tolerance was framed as a compromise rather than a change in sensory preference.

### 4.4 Object elicitation as a method for accessing tacit sensory judgement

Methodologically, this study demonstrates the value of object-elicitation workshops for accessing tacit sensory knowledge. Participants’ accounts suggested difficulty articulating preferences abstractly, with most relying instead on immediate evaluative phrases such as “it just feels right” (I2). Such expressions signalled judgements that were grounded in direct interaction with objects rather than in explanatory detail.

Handling cutlery allowed participants to reflect in action, linking bodily sensation to judgement without requiring technical sensory vocabulary. The combination of ranking tasks, written rationales, and group discussion enabled triangulation between immediate reactions and more reflective accounts. Using a framework approach supported systematic analysis while remaining grounded in concrete objects and features. This preserved the specificity of sensory description necessary for design translation.

Overall, object elicitation appears to be particularly useful in clinical contexts where experiential knowledge is embodied, situated, and difficult to verbalise.

### 4.5 Clinical implications

Although the study was exploratory and based on a modest single-site sample, the present findings offer preliminary indications that may be relevant for ED services adopting sensory-informed or neurodivergent-affirming approaches to care. Participants’ accounts suggest that cutlery can function as part of the sensory environment of eating, with the potential to shape comfort, predictability, and ease of engagement with mealtimes. In clinical settings where mealtimes are already associated with anxiety and reduced autonomy (37–39), attention to seemingly minor environmental factors may help reduce avoidable sensory distress. Allowing flexibility in cutlery choice, permitting patients to use personally preferred cutlery where feasible, and avoiding materials that are widely experienced as aversive may represent low-cost adjustments with potential benefits across treatment goals, including reduced mealtime distress, improved meal completion, lower cognitive load during eating, and, where relevant, weight restoration. These adaptations align with trauma-informed and person-centred models of care, which emphasise predictability, autonomy, and environmental sensitivity (8, 14, 20–22, 39).

The impact of using non-preferred cutlery extends beyond negative subjective experience and could plausibly affect aspects of treatment engagement. Managing sensory discomfort draws on cognitive and emotional resources that might support treatment engagement, skill development, and emotion regulation (40). In already demanding clinical contexts, avoidable sensory stressors of this kind may therefore add to overall load during recovery, although this proposition warrants further study in larger and more diverse samples (12, 41).

Cutlery preferences are also subject to interpretation within clinical practice. In many ED treatment contexts, preferences for specific cutlery are routinely framed as ED-driven rituals to be challenged, rather than as potentially legitimate sensory needs (40). While ED-related factors may contribute, for example, preference for smaller spoons may reflect concerns about portion size as well as mouth fit, these explanations are not mutually exclusive. Sensory and ED-related drivers may co-exist within the same preference. However, defaulting to a solely ‘disordered ritual’ framing risks pathologising authentic sensory experiences, particularly in neurodivergent patients (40). Clinicians should therefore consider sensory and ED-related explanations in parallel, rather than as competing accounts.

These findings tentatively suggest that recognising shared areas of sensory constraint while preserving individual choice may be more useful than imposing a universal cutlery standard. Within inpatient and day programme settings, incorporating cutlery preferences into broader sensory assessments may support more personalised mealtime planning without requiring substantial structural change. These propositions are offered as exploratory and would benefit from evaluation in larger and more diverse samples before being translated into service-level recommendations. Beyond the clinical environment, these findings are also relevant for behavioural experiments involving eating in real-world settings, such as canteens, cafes, or restaurants. For some individuals, bringing personally preferred cutlery can reduce sensory disruption and enable participation in social eating outside treatment. This adaptation does not represent a limitation but a strategy for full participation in social eating, consistent with disability justice principles that prioritise self-determination and accommodation over conformity to normative eating practices (42). Acknowledging and planning for sensory factors may therefore support graded exposure while affirming that equal participation can take different forms.

Finally, these recommendations should be considered within clinical constraints. Many ED settings operate within safety protocols that restrict access to certain utensils, particularly in acute inpatient care, where risk of self-harm or other safeguarding concerns may necessitate standardised ‘safe’ cutlery (43). Hygiene, infection control, and resource constraints also limit the range of materials that can realistically be offered. We therefore frame our findings as a call for flexibility within risk-appropriate boundaries. Incorporating cutlery preference into individualised sensory planning, where clinically feasible, is likely to be more achievable than system-wide redesign.

### 4.6 Design implications

These findings have implications for product design, clinical practice, and environmental adaptation within ED services. They provide a set of empirically grounded constraints and priorities for future design work. Key implications include:

- Avoiding wooden and paper-based materials in contexts where sensory sensitivity is likely
- Prioritising metal for its weight, stability, predictability, and ease of cleaning
- Avoiding hybrid designs such as sporks, which blur functional boundaries between cutlery types and may introduce additional sensory and hygiene concerns
- Attending to weight distribution and perceived solidity, avoiding overly light or flexible items
- Refining specific features, particularly spoon depth, width, and mouth fit
- Reducing seams, ridges, uneven transitions, and abrupt changes in thickness

These findings suggest that future prototyping should focus on controlled variation within acceptable sensory ranges rather than radical redesign. Design efforts should remain sensitive to individual differences while respecting shared sensory limits.

### 4.7 Strengths and limitations

This study has several strengths. To our knowledge, it is the first empirical investigation to examine cutlery as a sensory mediator within ED treatment. The findings contribute to the current understanding of sensory influences on eating experiences in ED contexts and provide foundational evidence to inform future prototyping of sensory-informed cutlery (14, 41). The use of structured object-elicitation workshops enabled direct, embodied engagement with the stimulus set, allowing access to sensory judgements that may not emerge through interviews or questionnaires. The mixed-methods design supported integration of descriptive preference patterns with experiential accounts and strengthened the overall interpretation.

Several limitations should be acknowledged. Participation was voluntary, introducing potential self-selection bias. The sample size was modest (n=24), and the study was exploratory; quantitative findings should therefore be interpreted descriptively.

The study was conducted within a single ED specialist service, which may limit transferability to other settings, age groups, or cultural contexts. The sample was entirely White and predominantly female (91.6%), limiting applicability to more ethnically and gender-diverse populations. Metal cutlery is the most common utensil type in everyday use in the UK, and preferences observed in this sample may therefore partly reflect familiarity rather than sensory properties alone. The study design does not allow these influences to be fully disentangled. These findings may not transfer to cultural contexts where other eating utensils are more commonly utilised (15).

Although autism and ADHD diagnoses were recorded, the study was not designed to examine differences by neurodivergent profile, and findings cannot be attributed to specific diagnostic groups. We also did not systematically assess for ARFID or ARFID traits, which may shape sensory-related cutlery preferences (44). Future research should include more diverse samples and explicitly examine cutlery preferences across neurodivergent and ARFID populations.

Finally, the stimulus set, while purposively varied, represented only a subset of possible cutlery designs. Cutlery was also handled without food, which allowed focused assessment of material and shape, but may not fully reflect experiences during actual meals, particularly under conditions of heightened sensory load or stress. The findings, therefore, reflect responses within the sampled design space rather than definitive conclusions about all cutlery types. Future research should therefore examine cutlery use during eating itself.

## 5 Conclusion

This study examined how adults receiving ED treatment evaluate everyday cutlery through structured sensory object elicitation. The findings demonstrate that while individuals differ in their preferences, there are shared sensory limits that shape what feels acceptable, and that cutlery functions as an active sensory interface during eating. Participants evaluated cutlery through embodied judgements of weight, balance, surface continuity, mouth fit, and material predictability.

While certain materials and designs were widely rejected (e.g., wooden and hybrid forms), preferences varied within what we term an *acceptable sensory range*: the bounded set of materials, weights, and features that participants in this sample did not consistently reject. In this sample, this included metal cutlery with adequate weight and smooth surface continuity. This concept is intended as an empirical guide for future development rather than a universal standard.

The study identified design-relevant insights grounded in lived sensory experience. These findings provide an empirical foundation for future iterative prototyping and co-design, particularly through feature-level refinement of spoons and other cutlery types. Future research should extend this work into real eating contexts, including meals involving food and varying levels of stress, to examine how sensory preferences interact with situational demands.

Grounding future design exploration in empirically identified sensory constraints may support the development of eating environments and tools that are both acceptable and usable within sensory-informed and neurodivergent-affirming ED care.

## Data Availability

The raw data supporting the conclusions of this article will be made available by the authors, without undue reservation.

## References

[1] Bell K Coulthard H Wildbur D . Self‐disgust within eating disordered groups: Associations with anxiety, disgust sensitivity and sensory processing. Eur Eating Disord Rev. (2017) 25:373–80. doi: 10.1002/erv.2529 28635077

[2] Kinnaird E Dandil Y Li Z Smith K Pimblett C Agbalaya R . Pragmatic sensory screening in anorexia nervosa and associations with autistic traits. J Clin Med. (2020) 9:1182. doi: 10.3390/jcm9041182 32326069 PMC7230430

[3] Brand-Gothelf A Parush S Eitan Y Admoni S Gur E Stein D . Sensory modulation disorder symptoms in anorexia nervosa and bulimia nervosa: A pilot study. Int J Eating Disord. (2016) 49:59–68. doi: 10.1002/eat.22460 26354076

[4] Merwin RM Moskovich AA Wagner HR Ritschel LA Craighead LW Zucker NL . Emotion regulation difficulties in anorexia nervosa: Relationship to self-perceived sensory sensitivity. Cognit Emot. (2013) 27:441–52. doi: 10.1080/02699931.2012.719003 22963392 PMC3593757

[5] Zucker NL Merwin RM Bulik CM Moskovich A Wildes JE Groh J . Subjective experience of sensation in anorexia nervosa. Behav Res Ther. (2013) 51:256–65. doi: 10.1016/j.brat.2013.01.010 23523866 PMC3955712

[6] Cassidy E Allsopp M Williams T . Obsessive compulsive symptoms at initial presentation of adolescent eating disorders. Eur Child Adolesc Psychiatry. (1999) 8:193–9. doi: 10.1007/s007870050129 10550701

[7] Makin L Meyer A Mondelli V Tchanturia K . Regulating with food: a qualitative study of neurodivergent experiences in adults with binge eating disorder. J Eating Disord. (2026) 14:13. doi: 10.1186/s40337-025-01493-7 41366481 PMC12801551

[8] Makin L Meyer A Chubinidze D Mondelli V Tchanturia K . Bingeing as an ADHD-related strategy: a qualitative study of experiences of neurodivergent and potentially neurodivergent adults with bulimic-spectrum eating disorders. Eating Weight Disorders-Studies Anorexia Bulimia Obes. (2025) 31(1):3. doi: 10.1007/s40519-025-01804-6 41329419 PMC12774937

[9] Kinnaird E Stewart C Tchanturia K . Taste sensitivity in anorexia nervosa: A systematic review. Int J Eating Disord. (2018) 51:771–84. doi: 10.1002/eat.22886 29984498 PMC6282513

[10] Kinnaird EM Isis Smith K Tchanturia K . Sensory sensitivities: Assessing, understanding and adapting. In: Tchanturia K , editor.Supporting autistic people with eating disorders: A guide to adapting treatment and supporting recovery. London: Jessica Kingsley Publishers (2021). p. 272.

[11] Kinnaird E Norton C Pimblett C Stewart C Tchanturia K . Eating as an autistic adult: An exploratory qualitative study. PloS One. (2019) 14:e0221937. doi: 10.1371/journal.pone.0221937 31465510 PMC6715205

[12] Nimbley E Golds L Sharpe H Gillespie-Smith K Duffy F . Sensory processing and eating behaviours in autism: A systematic review. Eur Eating Disord Rev. (2022) 30:538–59. doi: 10.1002/erv.2920 35737818 PMC9545673

[13] Petitpierre G Luisier A-C Bensafi M . Eating behavior in autism: Senses as a window towards food acceptance. Curr Opin Food Sci. (2021) 41:210–6. doi: 10.1016/j.cofs.2021.04.015 38826717

[14] Chubinidze D Zesch E Sarpong A Li Z Baillie C Tchanturia K . The sensory landscape and embodied experiences in anorexia nervosa treatment: An inpatient sensory ethnography. J Clin Med. (2024) 13:7172. doi: 10.3390/jcm13237172 39685631 PMC11642696

[15] Rippington N . Cutlery: The Science and Sensory Experience. In: Culinary Psychology: Food Choices and the Mind-Body Connection. Bicester: Karnac Books (2026). p. 179.

[16] Piqueras-Fiszman B Spence C . Do the material properties of cutlery affect the perception of the food you eat? An exploratory study. J Sens Stud. (2011) 26:358–62. doi: 10.1111/j.1745-459x.2011.00351.x 40046247

[17] Michel C Velasco C Spence C . Cutlery matters: Heavy cutlery enhances diners’ enjoyment of the food served in a realistic dining environment. Flavour. (2015) 4:26. doi: 10.1186/s13411-015-0036-y 38164791

[18] Harrar V Spence C . The taste of cutlery: How the taste of food is affected by the weight, size, shape, and colour of the cutlery used to eat it. Flavour. (2013) 2:21. doi: 10.1186/2044-7248-2-21 38164791

[19] Guthrie S Baker J Cahill J Hemsley B . Patient perspectives on inpatient mealtimes: Insights on swallowing, mental wellbeing and recovery. Int J Ment Health Nurs. (2026) 35:e70212. doi: 10.1111/inm.70212 41489069 PMC12766665

[20] Williams KL Dumont RL Schiano NR Lawlor KF Greaney K Kim R . Use of sensory adaptive environments with autistic children: A scoping review. Res Autism Spectr Disord. (2024) 114:102362. doi: 10.1016/j.rasd.2024.102362 38826717

[21] Li Z Holetic V Webb J Chubinidze D Byford S Tchanturia K . In-person and online sensory wellbeing workshop for eating disorders: Updated case series. J Eating Disord. (2023) 11:117. doi: 10.1186/s40337-023-00834-8 37443135 PMC10347786

[22] Moberg J . A call for a trauma-informed approach during compulsory care for enduring anorexia nervosa with combined PTSD–an autoethnographic perspective. J Eating Disord. (2025) 13:92. doi: 10.1186/s40337-025-01283-1 40426264 PMC12107862

[23] Sandy KJ Chernecki LL Leichner PP . Eating disorder patients’ opinions of cafeteria-style vs hospital-style presentation of meals. J Am Dietetic Assoc. (2007) 107:376–8. doi: 10.1016/j.jada.2007.01.018 17324652

[24] Brown C Tollefson N Dunn W Cromwell R Filion D . The adult sensory profile: Measuring patterns of sensory processing. Am J Occup Ther. (2001) 55:75–82. doi: 10.5014/ajot.55.1.75 11216370

[25] Tchanturia K Chubinidze D Duffy F Nimbley E Li Z Holliday J . Implementation insights from the PEACE pathway across UK eating disorder services. Nutrients. (2025) 17:1532. doi: 10.3390/nu17091532 40362840 PMC12073639

[26] Kahlke R Maggio LA Lee MC Cristancho S LaDonna K Abdallah Z . When words fail us: An integrative review of innovative elicitation techniques for qualitative interviews. Med Educ. (2025) 59:382–94. doi: 10.1111/medu.15555 39412120 PMC11906277

[27] Orr ER Ballantyne M Gonzalez A Jack SM . Visual elicitation: Methods for enhancing the quality and depth of interview data in applied qualitative health research. Adv Nurs Sci. (2020) 43:202–13. doi: 10.1016/j.jada.2007.01.018 32732605

[28] Tchanturia K Baillie C Biggs C Carr A Harrison A Li Z . Sensory wellbeing workshops for inpatient and day-care patients with anorexia nervosa. Neuropsychiatrie. (2022) 36:51–9. doi: 10.1007/s40211-021-00392-y 34129196 PMC8204121

[29] Gale NK Heath G Cameron E Rashid S Redwood S . Using the framework method for the analysis of qualitative data in multi-disciplinary health research. BMC Med Res Methodol. (2013) 13:117. doi: 10.1186/1471-2288-13-117 24047204 PMC3848812

[30] Ritchie J Spencer L . Qualitative data analysis for applied policy research. In: Analyzing Qualitative Data. London: Routledge (2002). p. 173–94.

[31] Lavis A . Food, bodies, and the “stuff” of (not) eating in anorexia. Gastronomica. (2016) 16:56–65. doi: 10.1525/gfc.2016.16.3.56 33021500

[32] Lavender A Shubert I de Silva P Treasure J . Obsessive-compulsive beliefs and magical ideation in eating disorders. Br J Clin Psychol. (2006) 45:331–42. doi: 10.1348/014466505x53579 17147100

[33] Rozin P Fallon AE . A perspective on disgust. psychol Rev. (1987) 94:23. doi: 10.1037/0033-295x.94.1.23 3823304

[34] Cisler JM Olatunji BO Lohr JM . Disgust, fear, and the anxiety disorders: A critical review. Clin Psychol Rev. (2009) 29:34–46. doi: 10.1016/j.cpr.2008.09.007 18977061 PMC2895912

[35] Troop NA Treasure JL Serpell L . A further exploration of disgust in eating disorders. Eur Eating Disord Rev. (2002) 10:218–26. doi: 10.1002/erv.444 41531421

[36] Spence C . Making sense of touch: A multisensory approach to the perception of objects. In: The Power of Touch. London: Routledge (2016). p. 45–61.

[37] Komarova D Chambers K Foye U Jewell T . Patient and clinician perspectives on supported mealtimes as part of anorexia nervosa treatment: A systematic review and qualitative synthesis. Eur Eat Disord Rev. (2024) 32:731–47. doi: 10.1002/erv.3081 38466637

[38] Lloyd EC Powell C Schebendach J Walsh BT Posner J Steinglass JE . Associations between mealtime anxiety and food intake in anorexia nervosa. Int J Eat Disord. (2021) 54:1711–6. doi: 10.1002/eat.23589 34323297 PMC8434846

[39] Healey H . The loss of autonomy in eating disorder treatment: A patient perspective. J Psychiatr Ment Health Nurs. (2025) 32:482–6. doi: 10.1111/jpm.13132 39485021 PMC11891432

[40] Loomes R Chivers K Georgeaux-Healy C Mandy W Jewell T . Understanding the autistic experience of restrictive eating disorders—a systematic review and qualitative‐synthesis. Eur Eating Disord Rev. (2025) 33:800–14. doi: 10.1002/erv.3181 40042436 PMC12171672

[41] Cobbaert L Hay P Mitchell PB Roza SJ Perkes I . Sensory processing across eating disorders: A systematic review and meta-analysis of self-report inventories. Int J Eat Disord. (2024) 57:1465–88. doi: 10.1002/eat.24184 38511825

[42] Tchanturia K Dandil Y Li Z Smith K Leslie M Byford S . A novel approach for autism spectrum condition patients with eating disorders: Analysis of treatment cost‐savings. Eur Eating Disord Rev. (2021) 29:514–8. doi: 10.1002/erv.2760 32648631

[43] Dodd DR Forrest LN Velkoff EA Smith AR . Managing suicidality and nonsuicidal self-injury in eating disorder treatment. Psychiatr Clinics. (2026) 49:67–82. doi: 10.1016/j.psc.2025.08.005 41708269

[44] Calisan Kinter R Ozbaran B Inal Kaleli I Kose S Bildik T Ghaziuddin M . The sensory profiles, eating behaviors, and quality of life of children with autism spectrum disorder and avoidant/restrictive food intake disorder. Psychiatr Q. (2024) 95:85–106. doi: 10.1007/s11126-023-10063-6 38085408

